# Predictors of Interbody Fusion and Adjacent Segment Disease Following Anterior Lumbar Interbody Fusion for Degenerative Pathologies

**DOI:** 10.3390/jcm15103636

**Published:** 2026-05-09

**Authors:** Zach Pennington, Abdelrahman M. Hamouda, Stanley Dennison, Michael L. Martini, Derrick Obiri-Yeboah, Jana Khalifeh, Rawad Turko, Mohamed M. El-Gohary, Clare A. Fogelson, Michelle J. Clarke, William E. Krauss, Brett A. Freedman, Melvin D. Helgeson, Ahmad N. Nassr, Arjun S. Sebastian, Anthony L. Mikula, Benjamin D. Elder

**Affiliations:** 1 Department of Neurosurgery, Mayo Clinic, Rochester, MN 55905, USA; 2 Department of Orthopaedic Surgery, Mayo Clinic, Rochester, MN 55905, USA

**Keywords:** ALIF, mechanical complications, subsidence, lumbar fusion, BMP, circumferential fusion

## Abstract

**Background/Objectives**: To identify predictors of successful fusion and adjacent segment disease (ASD) following ALIF. **Methods**: Records of patients undergoing one- or two-level ALIF were queried for baseline and postoperative radiographic data, demographics, operative notes, and implant characteristics. All had ≥1 year of follow-up with CT, and multivariable Cox regression was used to identify predictors of radiographic fusion through the interbody, ASD, and ASD requiring reoperation. **Results**: In total, 177 patients (median 59 yr; 52.5% male) were treated at 245 unique levels, of which 193 fused (81.3% with posterior fixation and 59.6% with standalone), 43 had ASD (17.6%), and 14 had ASD requiring reoperation (5.7%). Fusion was predicted by anterior cage placement (HR 0.94/mm; 95% CI [0.90, 0.98]; *p* = 0.003) and BMP use (HR 1.92; [1.15, 3.18]; *p* = 0.012). Radiographic ASD was predicted by older age (HR 1.08 per year; [1.03, 1.14]; *p* < 0.001), undergoing a revision [vs. index] fusion operation (HR 3.51; [1.44; 8.59]; *p* = 0.006), lower preoperative disc height (HR 0.83/mm; [0.74, 0.94]; *p* = 0.003), and preoperative facet vacuum phenomenon (HR 2.46; [1.18, 5.15]; *p* = 0.017). None of the extracted variables predicted reoperation for ASD. **Conclusions**: BMP use along with anterior cage placement and posterior fixation may improve the odds of fusion through the interbody following one- or two-level ALIF. Adjacent segment pathology is more common in patients with greater preoperative degenerative pathology (vacuum sign; more collapsed disc) and advanced age. Pelvic fixation did not improve fusion odds, but the data highlight the benefits of supplementary posterior fixation vs. standalone ALIF.

## 1. Introduction

Anterior lumbar interbody fusion (ALIF) is a powerful technique for achieving indirect decompression and segmental correction in the lumbar spine [1]. Additionally, by allowing the placement of a large graft with an accordingly large surface area, ALIF can reduce subsidence rates relative to posterior (PLIF) and transforaminal lumbar interbody fusion (TLIF) [2,3]. It also serves as an effective revision option for patients with prior failed posterolateral lumbar fusion [4]. Last, ALIF increases construct stability at the lumbosacral junction, which may reduce sacral screw strain [5] and the risk of rod breakage [6].

However, use of ALIF does not guarantee radiographic fusion and by increasing lumbar spine rigidity, it may increase stresses on the adjacent facet joints, precipitating adjacent segment degeneration (ASD) and the need for surgical revision [2]. The degree to which implant characteristics, baseline radiographic alignment, and surgical technique influence both pseudoarthrosis and ASD risk remains a point of ongoing investigation. In the present study we sought to investigate the relative contribution of these risk factors to pseudoarthrosis, radiographic ASD, and ASD requiring surgical revision.

## 2. Materials and Methods

After obtaining IRB approval (22-013333), we queried the medical record for patients who underwent 1- or 2-level ALIF as part of a standalone or combined anterior/posterior lumbar fusion at a single institution over a 46-month period. A waiver of consent was granted given the retrospective nature of this study and minimal risk to included patients. After identifying candidate patients, records were screened to identify those meeting inclusion/exclusion criteria. Patients were included if: (1) they underwent 1- or 2-level ALIF for degenerative pathologies; (2) had an upper instrumented vertebra (UIV) at or below L3; and (3) had minimum 1-year radiographic follow-up with postoperative CT scan to assess for radiographic fusion. We excluded patients if: (1) they underwent interbody fusion via PLIF, TLIF, or extreme/direct-lateral lumbar interbody fusion (XLIF/DLIF); (2) were treated for trauma, infection, or tumor; (3) lacked pre- or postoperative upright radiographs; or (4) lacked operative notes detailing used implants and the employed surgical technique.

**Figure 1:**
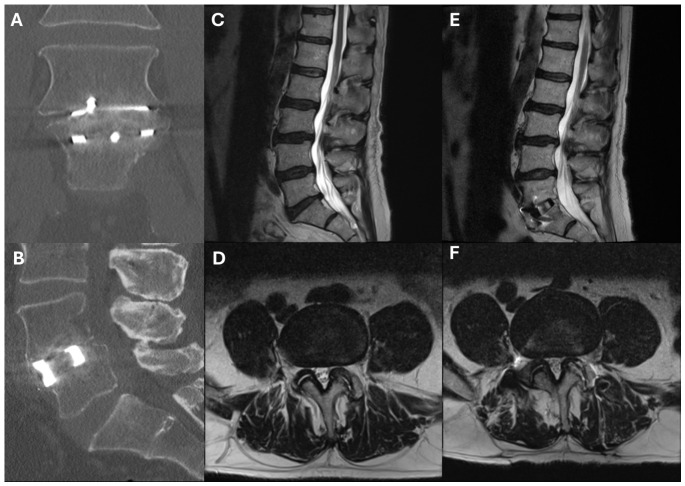
Illustration of outcomes of interest. (**A**,**B**) CT demonstrating formation of bridging bone through the interbody. (**C**–**F**) Comparison of preoperative (**C**,**D**), and postoperative (**E**,**F**) T2-weighted MR showing degeneration of the L4/5 disc in a patient who underwent L5-S1 ALIF. The patient ultimately required operative revision for symptomatic L4/5 adjacent segment disease.

Included patient records were then reviewed to extract data on patient demographics, surgery characteristics (treated level, construct length), implant properties (dimension, composition), pre- and postoperative upright radiographic alignment, and postoperative radiographic outcomes. We additionally queried whether the patient had prior surgery at the treated level, including a decompression procedure or instrumented fusion without interbody placement. The primary outcomes of interest were radiographic fusion on CT (bridging bone through interbody spacer), the occurrence of radiographic ASD, and the occurrence of ASD requiring operative revision (Figure 1). Radiographic ASD was defined by the presence of significant disc height loss, facet arthropathy, new listhesis or any other cause of spinal instability. Radiographic alignment parameters were measured on upright lumbar spine X-rays pre- and postoperatively (standing, prior to hospital discharge). Measured parameters included pelvic tilt (PT), pelvic incidence (PI), sacral slope (SS), L1-S1 lumbar lordosis (LL), L4-S1 LL, intradiscal lordosis (IDL) and disc height. Patient bone health was assessed using Hounsfield units on preoperative CT. These were measured as previously described [7,8] using regions of interest (ROIs) on axial CT slices through the cranial, mid-body, and caudal portion of the vertebra of interest. ROIs were placed in the cancellous bone and the three ROIs for each vertebral body were averaged. The patient’s paraspinal soft tissue envelope was also assessed using preoperative MRI. Parameters of interest were total cross-sectional area (CSA) of the multifidus and paraspinal muscles at L3, relative CSA of the multifidus and paraspinal muscles at L3 (defined as total CSA of the muscle belly divided by the CSA of the L3 mid-vertebral body), and fatty infiltration of the multifidus and psoas muscles, as assessed using the Goutallier scale [9].

### Statistical Analysis

Data were collected using Microsoft Excel (Redmond, WA, USA) and analyzed using IBM SPSS version 28.0.0 (Armonk, NY, USA). Descriptive statistics are reported as the mean and interquartile range for continuous data and counts with percentages for discrete data. Univariable analyses for the outcomes of interest were performed using Mann–Whitney U tests for continuous variables, Fisher exact tests for dichotomous variables, and χ^2^ analyses for categorical variables. Variables significant at the *p* < 0.05 level on univariable analyses were included in the multivariable analysis. To account for differences in the timing of the 1-year follow-up CT, multivariable Cox regression was performed to identify independent predictors of the outcome of interest. Variables highlighted as significant at the *p* < 0.05 level on univariable testing were eligible for inclusion in the multivariable model, which was constructed using forward stepwise selection with a threshold of *p* ≤ 0.05 for inclusion in the multivariable model and *p* > 0.10 for subsequent exclusion from the model. Evaluation for multicollinearity was performed by examining the coefficients of the linear regressions between included variables, looking for pairs of highly correlated variables. For all statistical tests, significance was defined as *p* < 0.05. The results of the Cox regression are presented as hazard ratios (HRs) with associated 95% confidence intervals (95% CIs).

## 3. Results

We identified 252 candidate patients (median 57 yr; 51.2% male) of whom 177 had sufficient radiographic follow-up (median 59 yr; 52.5% male) treated at 245 unique levels. The most commonly treated levels were L5/S1 (n = 154; 62.9%) and L4/5 (n = 86; 35.1%); 5 patients were implanted at L3/4 (2.0%). Most (76.7%) levels were implanted as part of a circumferential fusion construct and 60.4% of implants were titanium. Overall, 193 levels showed successful radiographic fusion (78.8%), 43 showed ASD (17.6%), and 14 required reoperation for ASD (5.7%).

### 3.1. Predictors of Radiographic Fusion

Univariable analysis (Table 1) showed that fused levels were more commonly implanted with polyetheretherketone (PEEK) [vs. titanium] interbodies (57.5% vs. 71.2%; *p* = 0.003), more commonly instrumented with combined anterior/posterior constructs (81.3% vs. 59.6%; *p* = 0.002), more commonly treated with BMP in the interbody (81.9% vs. 51.9%; *p* < 0.001), had lower preoperative PI (53.7° vs. 60.2°; *p* = 0.020) and disc height (5.4 mm vs. 6.4 mm; *p* = 0.047), lower postoperative PT (15.0° vs. 19.3°; *p* = 0.003), greater perioperative increase in segmental lordosis (11.8° vs. 9.9°; *p* = 0.026) and disc height (8.7 mm vs. 7.4 mm; *p* = 0.040), better apposition of the interbody to the anterior edge of the upper endplate (2.0 vs. 3.9 mm; *p* = 0.005), and lower paraspinal muscle CSA (3821 mm^2^ vs. 4311 mm^2^; *p* = 0.034). They were also more commonly instrumented to the pelvis (21.2% vs. 9.6%) though this was not significant (*p* = 0.071).

Multivariable analysis (Table 2) showed that poorer apposition of the interbody cage to the anterior apophyseal ring of the upper endplate (HR 0.940 per mm; 95% CI [0.903, 0.979]; *p* = 0.003) and use of BMP in the interbody (HR 1.915; [1.153, 3.182]; *p* = 0.012) were independent predictors of successful fusion through the interbody. Posterior fixation also approached significance (HR 1.613; [0.973, 2.674]; *p* = 0.064) and was included in the final model. Neither interbody material nor interbody footprint predicted radiographic fusion.

### 3.2. Predictors of Adjacent Segment Disease

Univariable analysis (Table 3) showed that levels with radiographic ASD by last follow-up were in older patients (63 yr vs. 57 yr; *p* < 0.001), more commonly had prior surgery at the treated level (34.9% vs. 17.3%; *p* = 0.013), had lower HU in the cranial vertebral body (126 vs. 143; *p* = 0.020), more commonly had vacuum phenomenon in one or both facets at the treated level (38.5 vs. 17.7%; *p* = 0.008), and had greater fatty degeneration of the multifidus muscle (Goutallier 3 vs. 2; *p* = 0.05). The segments showing ASD also had lower preoperative disc height (5.1 mm vs. 6.0 mm; *p* = 0.054), more commonly had a vacuum disc phenomenon (71.8% vs. 53.9%; *p* = 0.05), and more commonly had evidence of autofusion in one or both facets of the treated level (69.2% vs. 50.8%; *p* = 0.05), though these did not ultimately meet significance. On multivariable analysis (Table 4), the occurrence of radiographic ASD was predicted by increased age (HR 1.084 per year; [1.034, 1.136]; *p* < 0.001), undergoing revision [vs. index] surgery (HR 3.513; [1.437, 8.587]; *p* = 0.006), lower preoperative disc height (HR 0.830 per mm; [0.735, 0.938]; *p* = 0.003), and the presence of vacuum phenomenon in one or both facets at the treated level (HR 2.462; [1.176, 5.154]; *p* = 0.017). Increased multifidus Goutallier grade (HR 0.685 per grade; [0.450, 1.042]; *p* = 0.077) also approached significance and was included in the final model.

Similar univariable analyses were performed for ASD at 6-month, 1-year, 2-year, and 5-year follow-up. Only one level showed ASD by 6 months. At 12-month follow-up, levels showing ASD were associated with older age (73 vs. 60; *p* = 0.002), higher BMI (29.4 vs. 27.8; *p* = 0.036), lower preoperative SS (29.7 vs. 39.0; *p* = 0.024), and vacuum sign in one or both facets at the treated level (60% vs. 19.5%; *p* = 0.007). At 2-year follow-up, levels showing ASD were associated with older age (64 vs. 58; *p* < 0.001), shorter patient height (1.65 vs. 1.70 m; *p* = 0.028), lower HU in the upper vertebral body (116 vs. 142; *p* = 0.005) and upper endplate (305 vs. 386; *p* = 0.022), and the presence of vacuum phenomenon in one or both facets at the treated level (46.7 vs. 17.4%; *p* = 0.001). Last, at 5 years ASD was associated with older age (63 vs. 57; *p* < 0.001), lower HU of the upper vertebra (125 vs. 142; *p* = 0.024), greater Goutallier grade in the multifidus muscles (3 vs. 2; *p* = 0.028), and a greater prevalence of both prior surgery at the treated level (35.7% vs. 17.2%; *p* = 0.011) and vacuum sign in the facets of the treated level (39.5% vs. 17.6%; *p* = 0.005). Posterior fixation (*p* = 0.07), vacuum sign in the disc (*p* = 0.07) and autofusion of the facets (*p* = 0.07) also approached significance.

Univariable analysis of ASD requiring operative revision (Table 5) showed no significant differences between controls and levels requiring reoperation for ASD.

## 4. Discussion

Anterior lumbar interbody fusion is a popular technique for the management of pathologies in the lower lumbar spine. It allows the placement of a large interbody spacer, facilitates indirect foraminal decompression and direct decompression of discogenic pathologies, and preserves the posterolateral elements as a grafting surface in the case of circumferential fusion. However, it does not assure radiographic fusion and the stresses it places on adjacent motion segments can lead to accelerated adjacent segment disease [2]. In the present multivariable analyses, we noted the use of bone morphogenetic protein (BMP) in the interbody spacer and better apposition of the interbody spacer to the anterior edge of the cranial endplate were both significant predictors of successful radiographic fusion. The use of adjunct posterior fixation also approached significance and was included in the final model. Patient-specific parameters and interbody properties did not prove significant, highlighting the apparent primacy of surgical technique in influencing successful radiographic fusion. Interestingly, the use of PEEK vs. titanium spacers was associated with increased radiographic fusion on univariable analysis. However, after controlling for BMP use, placement of the interbody within the disc space, and posterior fixation, it proved not significant, likely owing to the higher rates of standalone ALIF in levels treated with titanium spacers. Additionally, when analyzing risk factors for radiographic ASD, revision surgery and radiographic evidence of greater baseline degeneration at the treated segment (vacuum phenomenon in the facets, lower disc height) were the best predictors of radiographic ASD. These factors did not prove predictive of ASD requiring reoperation though, highlighting the need for a better understanding of which factors predict the onset of symptoms in ASD and consequently influence the decision to perform revision surgery for ASD.

### 4.1. Predictors of Fusion Following ALIF

ALIF is currently the only FDA-approved indication for the use of BMP2, having been shown in a randomized controlled trial to produce similar rates of radiographic fusion relative to iliac crest autograft [10]. It has subsequently been demonstrated in several cohort studies to offer similar rates of radiographic fusion while reducing operative times [11]. Its association with increased odds of successful fusion in the present study is therefore unsurprising. However, BMP use is not entirely benign and has been associated with increased complications in some series. In one early systematic review, Singh and colleagues reported BMP use to be associated with increased risk of retrograde ejaculation, though it did not increase the overall rate of complications. In another systematic review examining the relation of BMP dose and surgical approach to complications [12], the authors noted graft subsidence, endplate resorption, and retrograde ejaculation as potential complications with the overall rate of complications increasing with BMP dose. However, when analyzed by specific complication, they noted the rates of retrograde ejaculation were highest in the control (non-BMP) and lowest BMP dose arms, and lowest in the higher does arms [12]. Consequently, while BMP use has been reported to increase the risk for retrograde ejaculation in male patients following ALIF, this review, among other series, suggests that it is an approach-related complication rather than a complication caused by BMP [13,14,15]. By contrast, the review authors [12] noted higher rates of graft subsidence and endplate resorption in the high dose arms (≥4.3 mg/level) without commensurate increases in the rate of radiographic fusion. The association of both endplate resorption and graft subsidence with high-dose BMP is likely due to the increased bone turnover caused by BMP administration. With high doses there can be local bone resorption, causing adjacent vertebral body osteolysis [16,17] and thus early implant subsidence. Over the same timeframe, it can also cause lumbar root radiculitis [18]. However, a recent single-center series [19] including 1095 anterior lumbar cases found BMP use to be associated with a lower rate of both novel and nonresolved radiculitis. More long-term BMP use can lead to heterotopic ossification, which has been reported to occur in approximately 14–20% of cases, though this seldom has clinical sequelae [20,21]. Current evidence also fails to support the notion that BMP has significant carcinogenic potential [22].

BMP does not ensure radiographic fusion. Even in the early investigational study by Burkus and colleagues [10], only 94.5% of patients treated with BMP achieved radiographic fusion by two-year follow-up. Similar rates have been reported by other, more contemporary studies, such as those of Galimberti et al. (97.8%) [23], Jowdy et al. (97.6%) [24], and Razzouk et al. (95.5%) [25]. Yet it is has been previously shown that BMP use significantly increases the direct costs of anterior lumbar interbody fusion [11]. For this reason, many cost-conscious surgeons seek to avoid its use, even in ALIF, unless the patient is noted to have significant risk factors for nonunion, such as prior surgery or a significant smoking history [26].

The use of a standalone ALIF vs. ALIF with posterior fixation may be another such risk factor. Despite the high reported rate of fusion in many of the contemporary ALIF studies, supplementary posterior fixation has been employed in many. It has been previously shown that standalone ALIF is associated with higher rates of pseudoarthrosis relative to ALIF with posterior fixation. One recent systematic review by Manzur and colleagues [26] noted successful fusion in only 89% of patients treated with standalone ALIF, though the rate increased to 94% with BMP2 use. By comparison, ALIF with posterior fixation has been reported to achieve radiographic fusion in 95–98% of cases, even in the absence of BMP use [27,28]. Data such as these beg the question of whether BMP use improves fusion rates following ALIF with posterior fixation. The present results suggest this is the case, as both BMP use and posterior fixation were independent predictors of successful radiographic fusion.

The addition of posterior fixation to ALIF may improve fusion odds by increasing baseline construct stability. Cadaveric work and finite element analysis [29] have shown that the addition of posterior fixation to ALIF results in a 69–92% reduction in range of motion. By facilitating load sharing between the pedicle screw construct and interbody, the addition of posterior fixation also decreases peak endplate stresses by 20–50% in finite element testing [29]. This reduces cage subsidence risk, which has been suggested in some studies to increase the rate of nonunion [30]. Clinically, Laiwalla and colleagues noted the addition of posterior fixation to reduce the risk of reoperation for nonunion in a multicenter study of patients undergoing ALIF between L4 and S1 [31]. Implant parameters were not considered and a multivariable analysis considering both BMP use and posterior fixation was not considered. Holte et al. [32] had similarly noted higher rates of fusion when posterior instrumentation was added to the anterior fusion construct. However, it was conducted prior to the advent of BMP. Anjarwalla et al. [33] and McCarthy et al. [34] likewise reported the addition of posterior fixation to improve the odds of radiographic fusion, though like in Holte et al. [32] and Laiwalla et al [31]., no multivariable analysis controlling for BMP use and posterior fixation was performed.

Interestingly, interbody fusion material was ultimately not a significant predictor of successful fusion in the present cohort. On univariable analysis, PEEK interbody use was associated with increased odds of successful fusion. This contradicts the majority of published studies, which suggest fusion outcomes are superior for titanium interbodies, likely due to the hydrophobic nature of PEEK [35]. However, PEEK interbodies were more commonly implanted with BMP2 in the graft and combined with posterior fixation, both of which were shown to predict increased odds of radiographic fusion. It therefore seems likely that our observed association is an artifact of the asymmetric use of BMP2 and posterior fixation. Furthermore, while we failed to observe a positive association of titanium interbody use with radiographic fusion, it may be that these results simply suggest interbody material choice is a less important determinant of successful fusion.

More recently it has been suggested that the local microenvironment may also play a strong role in fusion outcomes. Animal work has demonstrated that upregulation of pre-inflammatory factors, such as TNF-α, may inhibit bony fusion [36]. In their study of a rat model of posterolateral lumbar fusion, Ng and colleagues noted that inclusion of TNFα-secreting stem cells in the bone graft material was sufficient to prevent the formation of bridging bone on microCT and histologic sections. While the inflammatory phase is necessary to initiate bony healing [37], the authors conjectured that continued inflammation likely prevents transition to the reparative and remodeling stages necessary for fusion. It has been suggested that the poor affinity of the BMP2 collagen sponge carrier for BMP2 protein leads to a high initial concentration of BMP2 and a resultant supraphysiologic inflammatory response [38]. If sustained, such a response would likely negate the osteogenic and osteoinductive properties of BMP2 and could in part explain why it is insufficient to ensure radiographic fusion. To this end, Glaeser et al. [39] also employed a rat model of lumbar fusion and found that treatment of rats with BMP2 and NEMO binding domain (NBD) peptide, an inhibitor of the NF-κB-mediated immune response, reduced BMP2-induced soft tissue inflammation. There was also greater tighter bone trabecular spacing and greater bone formation in the NBD/BMP2-treated group relative to animals treated with BMP2 alone. However, at present there has been no translation into a human therapeutic.

### 4.2. ALIF and Adjacent Segment Disease

By increasing the stiffness of the implanted segment, ALIF, like any fusion operation, funnels stresses to the adjoining motion segments, which can precipitate adjacent segment disease. Here we considered both radiographic ASD and the more clinically relevant outcome of ASD requiring reoperation. ASD was associated with decreased preoperative disc height and facet joint vacuum phenomenon, both likely surrogates of a motion segment with greater baseline degeneration. Additionally, increased patient age, undergoing revision [vs. index] surgery, and greater multifidus fatty infiltration were also predictive of radiographic ASD. This may suggest that a globally more degenerated spine is less able to accommodate the biomechanical changes following instrumented fusion and hence is more prone to suffering ASD. This has been suggested by some other groups. In one study, Song and colleagues [40] compared rates of symptomatic degeneration at levels immediately adjacent to a fused level and levels not immediately adjacent to the fused level. While radiographic ASD was more common at the adjacent level, rates of symptomatic degeneration did not differ, suggesting that the symptomatic degeneration may be more representative of the natural history of the patient’s spine and therefore represent an already compromised spinal environment. Kim et al. similarly argued for this in their study comparing patients with ASD following posterior lumbar fusion to those without [41]. Those experiencing ASD had more atrophic paraspinal muscles, greater preoperative disc degeneration on MRI, and greater facet degeneration on CT, all arguing that those levels showing ASD were already more compromised. The altered biomechanics following fusion may therefore have simply added stress to an already compromised system, accelerating the degeneration. Such a hypothesis has seen support in finite element work, which has suggested that preoperative malalignment and muscle atrophy, as would be seen in a grossly degenerated spine, may contribute more significantly to the development of ASD than the fusion operation itself.

A handful of prior studies have looked at risk factors for ASD following ALIF. One study by Martini and colleagues considered 404 patients who underwent ALIF at one or two levels [42] and noted that reoperation for ASD was positively associated with increased age, increased BMI, increased medical comorbidities, and undergoing two- vs. one-level surgery. BMP use, the use of posterior fixation, or implant parameters were not considered, nor was the presence of preoperative signs of spondylotic changes or paraspinal muscle degeneration. The latter two were noted to be significant predictors of radiographic ASD in the present study. The study by Laiwalla et al. [31] also examined ASD requiring surgical revision following ALIF. Specifically, they evaluated the association of posterior fixation on operative ASD risk and noted no significant association. No other parameters were considered.

Interestingly, none of the risk factors for radiographic ASD were found to predict ASD requiring surgical revision. Though the reason for this is unclear, it does highlight the dissociation of radiographic and clinical phenomena. More recently, studies have noted that patients with ASD are often asymptomatic. One study by Mannion et al. [43] examined patient-reported outcomes (PROs) following lumbar fusion and noted no significant difference in PROs between patients that did and did not show evidence of radiographic ASD. Similarly, Liu et al. [44] compared PROs between patients with and without radiographic ASD following short-segment lumbar fusion and saw no significant difference. This inconsistent relationship between radiographic and clinical ASD may reflect the extremely broad definition normally employed for radiographic ASD [45], which includes pathologies that require revision surgery (e.g., proximal junctional failure), those that require surgical revision in the setting of symptoms (e.g., disc herniation cephalad UIV, degenerative spondylolisthesis) [46], and those that often do not require reoperation (e.g., facet arthropathy, disc desiccation) [47]. Consequently, while multiple studies have looked at ASD following lumbar fusion [48,49], further investigation of those parameters that precipitate symptomatic ASD or subanalysis of ASD by phenotype may be more useful for guiding clinical practice.

### 4.3. Limitations

There are several limitations to the present study. First, as a retrospective study, we are precluded from attributing any causality to the observed associations. Additionally, as a single-institution study, there may be unique institutional practices such as end plate preparation, interbody trial placement, and intraoperative disc space distraction technique, which are not more broadly practiced, limiting the generalizability of the observed results. To this end, there is also variation in operator experience wherein most surgeons contributing to the present series were ≥10 years into independent practice. This is not reflective of general practice and so may further bias the results. Also, we made the assessment of radiographic fusion based upon the presence of bridging bone on postoperative CT scans. While this is an accepted definition, other studies have employed bridging bone on plain films or interspinous process motion. These alternative definitions may alter the observed predictors of successful fusion. Furthermore, we performed the statistical analyses on a “by level” basis; however, in patients undergoing multilevel fusion, the levels are not truly independent. We attempt to account for this using the multivariable regression, but we are unable to fully account for this and the number of levels fused may therefore influence the risk of ASD or nonunion to a degree for which we cannot account. The present study is additionally limited by a relatively short follow-up. The minimum required for inclusion in the present study was 1 year, which may be insufficient to capture many cases of ASD, which is reported to occur at a rate of 2–3% per year following lumbar surgery [50]. Last, the two definitions for ASD employed here were radiographic ASD and ASD requiring reoperation. As the literature correlating ASD with worsened PROs is variable, it would be valuable for further research to be conducted looking at predictors of symptomatic ASD.

## 5. Conclusions

In the present study, we evaluated predictors of both radiographic fusion and adjacent segment disease following ALIF. BMP use and the placement of posterior fixation both appeared to increase rates of radiographic fusion. Surgical factors were largely unimportant in predicting adjacent segment disease, though. Rather more advanced motion segment degeneration, as signified by more advanced patient agent, facet joint vacuum phenomenon, and greater preoperative disc space collapse, appeared to best predict the occurrence of radiographic ASD, though they did not predict ASD requiring reoperation. Further investigation focusing on the correlation of patient symptom severity and the decision to undergo reoperation for adjacent segment disease is likely merited.

## Figures and Tables

**Table 1:** Comparison of levels with fusion through the interbody at 1 year to those without successful fusion.

Variable	Pseudarthrosis	Fusion	*p*
	52	193	
Demographics			
Age (yr)	60 [52, 63]	60 [46, 68]	0.63
Sex (female)	26 (50.0)	103 (53.4)	0.76
Height (m)	1.69 [1.63, 1.78]	1.70 [1.64, 1.80]	0.62
Weight (kg)	84.7 [72.3, 95.4]	82.2 [68.5, 97.2]	0.45
BMI (kg/m^2^)	27.6 [26.6, 32.7]	28.1 [24.5, 31.0]	0.33
Surgery Characteristics			
Construct length (levels)	2 [1, 2]	2 [1, 2]	0.05
Implant height (mm)	16.0 [14.0, 18.0]	15.0 [14.0, 17.0]	0.19
Implant width (mm)	36 [36, 40]	36 [34, 38]	0.32
Implant length (mm)	25 [24, 27]	25 [24, 28]	0.11
Implant lordosis (°)	16 [12, 18]	16 [14, 18]	0.71
Material			0.003
Titanium	37 (71.2)	111 (57.5)	
PEEK	13 (25.0)	82 (42.5)	
Allograft	2 (3.8)	0 (0)	
2-level surgery	25 (48.1)	112 (58.0)	0.21
Prior surgery at treated level	11 (21.2)	39 (20.2)	0.85
Spondylolisthesis at treated level	27 (51.9)	87 (45.1)	0.43
Implanted level			0.37
L3/4	0 (0)	5 (2.6)	
L4/5	21 (40.4)	65 (33.7)	
L5/S1	31 (59.6)	123 (63.7)	
Posterior instrumentation	31 (59.6)	157 (81.3)	0.002
BMP used in interbody	27 (51.9)	158 (81.9)	<0.001
Pelvic fixation	5 (9.6)	41 (21.2)	0.071
Preoperative Radiographics			
PI (°)	60.2 [52.6, 72.7]	53.7 [47.0, 66.6]	0.02
PT (°)	19.9 [14.1, 27.8]	18.4 [12.3, 22.7]	0.06
SS (°)	41.9 [35.4, 52.2]	37.9 [31.3, 44.8]	0.05
L1-S1 LL (°)	59.2 [50.9, 67.7]	54.9 [44.2, 64.3]	0.05
L4-S1 LL (°)	36.3 [28.6, 41.8]	31.3 [25.6, 38.5]	0.03
IDL (°)	7.7 [5.3, 12.6]	6.8 [3.0, 11.7]	0.21
Disc height (mm)	6.4 [4.6, 7.9]	5.4 [3.6, 7.6]	0.047
HU Upper Body	138 [111, 167]	139 [115, 176]	0.93
HU Lower Body	181 [138, 256]	183 [140, 229]	0.97
HU Upper Endplate	417 [296, 524]	364 [300, 470]	0.39
HU Lower Endplate	421 [271, 524]	405 [277, 517]	0.93
Vacuum disc at treated level	28 (53.3)	112 (58.1)	0.61
Vacuum phenomenon in facets	12 (22.7)	41 (21.0)	0.84
Facet autofusion	30 (57.8)	103 (53.2)	0.62
Anterior bridging osteophyte	3 (5.8)	10 (5.2)	0.72
Postoperative Radiographics			
PI (°)	59.8 [53.7, 67.8]	55.9 [46.9, 64.6]	0.027
PT (°)	19.3 [13.7, 28.3]	15.0 [10.5, 20.4]	0.003
SS (°)	42.9 [35.6, 50.3]	40.5 [32.3, 47.5]	0.32
L1-S1 LL (°)	56.7 [45.0, 66.2]	55.5 [45.7, 66.7]	0.56
L4-S1 LL (°)	45.0 [37.8, 50.6]	44.8 [37.0, 50.7]	0.81
IDL (°)	17.8 [14.2, 22.2]	19.9 [15.6, 23.4]	0.08
ΔIDL (°)	9.9 [4.7, 12.9]	11.8 [6.5, 16.1]	0.026
Disc height (mm)	14.3 [12.4, 16.4]	14.3 [12.7, 16.0]	0.98
ΔDisc height (mm)	7.4 [5.9, 10.3]	8.7 [6.5, 11.3]	0.040
Cage distance to apophyseal ring (mm)			
Anterior edge upper endplate	3.9 [0, 5.3]	2.0 [0, 4.2]	0.005
Anterior edge lower endplate	2.5 [0, 4.5]	1.7 [0, 3.9]	0.33
Posterior edge upper endplate	10.0 [4.0, 20.3]	11.3 [5.2, 28.8]	0.099
Posterior edge upper endplate	11.1 [5.8, 21.3]	12.4 [6.8, 29.6]	0.15
Morphometrics at L3			
Multifidus (absolute, mm^2^)	1015 [874, 1313]	1027 [823, 1294]	0.49
Multifidus (relative)	0.87 [0.69, 1.05]	0.86 [0.68, 1.10]	0.95
Paraspinals (absolute, mm^2^)	4311 [3396, 4756]	3821 [3063, 4568]	0.034
Paraspinals (relative)	3.51 [2.70, 4.21]	3.25 [2.67, 3.99]	0.21
Goutallier—multifidus	3 [2, 4]	2 [2, 4]	0.24
Goutallier—psoas	0 [0, 1]	0 [0, 1]	0.39

Key: HU—Hounsfield unit; IDL—intradiscal lordosis; LL—lumbar lordosis; mm—millimeter; PI—pelvic incidence; PT—pelvic tilt; SS—sacral slope.

**Table 2:** Multivariable Cox regression for predictors of fusion through the interbody.

Variable	HR	95% CI	*p*
Posterior fixation	1.613	[0.973, 2.674]	0.064
Distance cage to anterior edge upper endplate (per mm)	0.940	[0.903, 0.979]	0.003
BMP used in interbody	1.915	[1.153, 3.182]	0.012

Key: CI—confidence interval; HR—hazard ratio; mm—millimeter.

**Table 3:** Comparison of levels with radiographic adjacent segment disease by last follow-up to those without adjacent segment disease.

Variable	ASD	No ASD	*p*
	43	202	
Demographics			
Age (yr)	63 [57, 73]	57 [46, 66]	<0.001
Sex (female)	21 (48.8)	108 (53.5)	0.62
Height (m)	1.72 [1.63, 1.83]	1.70 [1.64, 1.79]	0.68
Weight (kg)	80.2 [70.2, 100.0]	84.0 [68.4, 96.0]	0.38
BMI (kg/m^2^)	28.4 [27.3, 29.6]	27.8 [24.5, 31.2]	0.31
Surgery Characteristics			
Construct length (levels)	2 [1, 2]	2 [1, 2]	0.93
Implant height (mm)	16.0 [14.0, 17.0]	16.0 [14.0, 17.0]	0.73
Implant width (mm)	36 [36, 40]	36 [34, 38]	0.08
Implant length (mm)	27 [24, 27]	25 [24, 28]	0.81
Implant lordosis (°)	16 [12, 18]	16 [14, 18]	0.37
Material			0.34
Titanium	30 (69.8)	118 (58.4)	
PEEK	13 (30.2)	82 (40.6)	
Allograft	0 (0)	2 (1.0)	
2-level surgery	26 (60.5)	111 (55.0)	0.61
Prior surgery at treated level	15 (34.9)	35 (17.3)	0.013
Spondylolisthesis at treated level	22 (51.2)	92 (45.5)	0.51
Implanted level			0.51
L3/4	0 (0)	5 (2.5)	
L4/5	14 (32.6)	72 (35.6)	
L5/S1	29 (67.4)	125 (61.9)	
Posterior instrumentation	38 (88.4)	150 (74.3)	0.049
BMP used in interbody	35 (81.4)	150 (74.3)	0.44
Pelvic fixation	8 (18.6)	38 (18.8)	>0.99
Preoperative Radiographics			
PI (°)	53.7 [46.7, 69.4]	55.4 [48.1, 67.0]	0.90
PT (°)	19.9 [11.8, 25.8]	18.3 [12.4, 23.2]	0.30
SS (°)	35.0 [31.0, 48.0]	39.0 [32.1, 45.3]	0.62
L1-S1 LL (°)	53.5 [48.0, 62.0]	56.8 [46.8, 65.2]	0.30
L4-S1 LL (°)	28.6 [22.6, 37.7]	32.7 [27.0, 39.9]	0.07
IDL (°)	5.6 [2.4, 9.3]	7.3 [3.6, 12.3]	0.08
Disc height (mm)	5.1 [3.7, 6.2]	6.0 [3.8, 7.8]	0.054
HU Upper Body	126 [99, 160]	143 [120, 178]	0.020
HU Lower Body	182 [130, 221]	183 [142, 231]	0.70
HU Upper Endplate	345 [274, 491]	383 [308, 491]	0.24
HU Lower Endplate	352 [277, 527]	419 [277, 514]	0.64
Vacuum disc at treated level	31 (71.8)	109 (53.9)	0.05
Vacuum phenomenon in facets	17 (38.5)	36 (17.7)	0.008
Facet autofusion	30 (69.2)	103 (50.8)	0.05
Anterior bridging osteophyte	4 (10.3)	9 (4.5)	0.23
Postoperative Radiographics			
PI (°)	57.6 [48.1, 69.8]	57.4 [47.8, 66.4]	0.59
PT (°)	19.0 [13.7, 21.8]	15.0 [11.1, 21.0]	0.12
SS (°)	40.0 [31.8, 46.7]	41.1 [33.7, 48.6]	0.58
L1-S1 LL (°)	55.0 [45.4, 66.2]	55.7 [46.0, 66.7]	0.86
L4-S1 LL (°)	45.2 [37.2, 50.3]	44.7 [37.8, 50.7]	0.61
IDL (°)	18.0 [13.9, 24.3]	19.4 [15.6, 23.1]	0.70
ΔIDL (°)	12.4 [7.8, 16.0]	11.2 [5.5, 15.7]	0.24
Disc height (mm)	14.1 [12.2, 15.3]	14.3 [12.7, 16.2]	0.33
ΔDisc height (mm)	8.9 [7.7, 10.4]	8.2 [6.2, 11.3]	0.27
Cage distance to apophyseal ring (mm)			
Anterior edge upper endplate	2.5 [0, 5.0]	2.5 [0, 4.8]	0.64
Anterior edge lower endplate	1.9 [−0.2, 4.0]	2.0 [0, 4.2]	0.48
Posterior edge upper endplate	9.8 [4.5, 15.4]	11.6 [5.2, 28.6]	0.14
Posterior edge upper endplate	9.8 [6.3, 16.6]	12.4 [6.9, 29.4]	0.17
Morphometrics at L3			
Multifidus (absolute, mm^2^)	1011 [827, 1273]	1021 [818, 1296]	0.69
Multifidus (relative)	0.86 [0.71, 1.05]	0.86 [0.68, 1.10]	0.92
Paraspinals (absolute, mm^2^)	3895 [3193, 4710]	3980 [3222, 4692]	0.76
Paraspinals (relative)	3.16 [2.65, 3.75]	3.40 [2.70, 4.06]	0.57
Goutallier—multifidus	3 [2, 4]	2 [1, 4]	0.050
Goutallier—psoas	0 [0, 1]	0 [0, 1]	0.56

Key: ASD—adjacent segment disease; HU—Hounsfield unit; IDL—intradiscal lordosis; LL—lumbar lordosis; mm—millimeter; PI—pelvic incidence; PT—pelvic tilt; SS—sacral slope.

**Table 4:** Multivariable Cox regression for predictors of adjacent segment disease following ALIF.

Variable	HR	95% CI	*p*
Age (per year)	1.084	[1.034, 1.136]	<0.001
Revision [index] operation	3.513	[1.437, 8.587]	0.006
Preoperative disc height (per mm)	0.830	[0.735, 0.938]	0.003
Goutallier grade—multifidus	0.685	[0.450, 1.042]	0.077
Vacuum phenomenon in facets	2.462	[1.176, 5.154]	0.017

Key: CI—confidence interval; HR—hazard ratio; mm—millimeter.

**Table 5:** Comparison of levels with adjacent segment disease requiring reoperation by last follow-up to those without adjacent segment disease.

Variable	ASD	No ASD	*p*
	14	231	
Demographics			
Age (yr)	63 [60, 63]	59 [48, 67]	0.12
Sex (female)	7 (50.0)	122 (52.8)	>0.99
Height (m)	1.72 [1.68, 185]	1.70 [1.64, 1.79]	0.22
Weight (kg)	84.2 [82.2, 98.1]	83.4 [68.5, 97.2]	0.41
BMI (kg/m^2^)	28.4 [27.6, 29.5]	27.9 [24.5, 31.6]	0.63
Surgery Characteristics			
Construct length (levels)	2 [1, 2]	2 [1, 2]	0.93
Implant height (mm)	15.5 [12.0, 18.0]	16.0 [14.0, 17.0]	0.85
Implant width (mm)	39 [36, 40]	36 [34, 38]	0.06
Implant length (mm)	27 [26, 28]	25 [24, 28]	0.06
Implant lordosis (°)	16 [12, 18]	16 [12, 18]	0.72
Material			0.91
Titanium	9 (64.3)	139 (60.2)	
PEEK	5 (35.7)	90 (39.0)	
Allograft	0 (0)	2 (0.9)	
2-level surgery	6 (42.9)	131 (56.7)	0.41
Prior surgery at treated level	5 (35.7)	45 (19.5)	0.17
Spondylolisthesis at treated level	5 (35.7)	109 (47.2)	0.58
Implanted level			0.43
L3/4	0 (0)	5 (2.2)	
L4/5	3 (21.4)	83 (35.9)	
L5/S1	11 (78.6)	143 (61.9)	
Posterior instrumentation	11 (78.6)	177 (76.6)	>0.99
BMP used in interbody	11 (78.6)	174 (75.3)	>0.99
Pelvic fixation	3 (21.4)	43 (18.6)	0.73
Preoperative Radiographics			
PI (°)	60.2 [48.0, 67.8]	54.9 [48.0, 67.0]	0.84
PT (°)	18.0 [13.0, 20.7]	18.6 [12.4, 23.6]	0.93
SS (°)	44.1 [32.2, 48.8]	38.0 [32.0, 45.1]	0.55
L1-S1 LL (°)	56.1 [50.3, 60.7]	56.0 [45.6, 65.2]	0.59
L4-S1 LL (°)	33.6 [27.9, 41.1]	32.2 [26.2, 39.7]	0.47
IDL (°)	7.5 [5.6, 13.5]	7.0 [3.4, 11.8]	0.48
Disc height (mm)	6.2 [4.8, 8.4]	5.6 [3.7, 7.6]	0.25
HU Upper Body	126 [121, 131]	140 [115, 175]	0.23
HU Lower Body	196 [151, 217]	182 [140, 230]	0.82
HU Upper Endplate	413 [299, 534]	367 [300, 487]	0.57
HU Lower Endplate	475 [244, 551]	400 [277, 514]	0.57
Vacuum disc at treated level	10 (72.7)	130 (56.3)	0.36
Vacuum phenomenon in facets	2 (16.7)	50 (21.6)	>0.99
Facet autofusion	6 (42.8)	126 (54.6)	0.76
Anterior bridging osteophyte	1 (9.1)	12 (5.3)	0.47
Postoperative Radiographics			
PI (°)	60.4 [48.2, 69.8]	57.4 [48.1, 66.3]	0.73
PT (°)	19.5 [16.4, 26.6]	15.9 [11.2, 21.0]	0.08
SS (°)	35.5 [31.9, 41.1]	41.2 [33.5, 48.6]	0.28
L1-S1 LL (°)	54.6 [52.2, 60.2]	55.7 [45.2, 66.7]	0.88
L4-S1 LL (°)	45.3 [41.1, 50.0]	44.8 [37.2, 50.7]	0.97
IDL (°)	17.9 [16.3, 21.6]	19.3 [14.9, 23.3]	0.83
ΔIDL (°)	9.4 [6.9, 13.4]	11.3 [5.8, 16.0]	0.51
Disc height (mm)	15.5 [13.5, 16.8]	14.3 [12.5, 15.9]	0.23
ΔDisc height (mm)	8.7 [7.6, 9.6]	8.4 [6.4, 11.2]	0.99
Cage distance to apophyseal ring (mm)			
Anterior edge upper endplate	2.3 [0, 4.2]	2.5 [0, 4.8]	0.92
Anterior edge lower endplate	1.5 [0, 3.9]	2.0 [0, 4.2]	0.76
Posterior edge upper endplate	10.7 [4.4, 12.9]	11.0 [5.0, 27.6]	0.53
Posterior edge upper endplate	9.3 [6.0, 13.9]	12.3 [6.7, 28.9]	0.40
Morphometrics at L3			
Multifidus (absolute, mm^2^)	1075 [836, 1928]	1016 [825, 1283]	0.29
Multifidus (relative)	0.80 [0.78, 1.55]	0.86 [0.68, 1.10]	0.55
Paraspinals (absolute, mm^2^)	3432 [3194, 4173]	3981 [3222, 4708]	0.38
Paraspinals (relative)	2.82 [2.65, 3.21]	3.41 [2.68, 4.06]	0.06
Goutallier—multifidus	2 [2, 4]	2 [2, 4]	0.91
Goutallier—psoas	0 [0, 1]	0 [0, 1]	0.44

Key: ASD—adjacent segment disease; HU—Hounsfield unit; LL—lumbar lordosis; mm—millimeter; PI—pelvic incidence; PT—pelvic tilt; IDL—intradiscal lordosis; SS—sacral slope.

## Data Availability

The data presented in this study are available on request from the corresponding author. The data are not publicly available due to privacy restrictions.

## Abbreviations

The following abbreviations are used in this manuscript:

ALIF	Anterior lumbar interbody fusion
ASD	Adjacent segment disease
CI	Confidence interval
DLIF	Direct lateral lumbar interbody fusion
HR	Hazard ratio
HU	Hounsfield unit
IDL	Intradiscal lordosis
LL	Lumbar lordosis
mm	Millimeter
PI	Pelvic incidence
PLIF	Posterior lumbar interbody fusion
PT	Pelvic tilt
SS	Sacral slope
TLIF	Transforaminal lumbar interbody fusion
XLIF	Extreme lumbar interbody fusion
